# *Plasmodium vivax* Malaria Endemicity in Indonesia in 2010

**DOI:** 10.1371/journal.pone.0037325

**Published:** 2012-05-17

**Authors:** Iqbal R. F. Elyazar, Peter W. Gething, Anand P. Patil, Hanifah Rogayah, Elvieda Sariwati, Niken W. Palupi, Siti N. Tarmizi, Rita Kusriastuti, J. Kevin Baird, Simon I. Hay

**Affiliations:** 1 Eijkman-Oxford Clinical Research Unit, Jakarta, Indonesia; 2 Spatial Ecology and Epidemiology Group, Department of Zoology, University of Oxford, Oxford, United Kingdom; 3 Directorate of Vector-Borne Diseases, Indonesian Ministry of Health, Jakarta, Indonesia; 4 Centre for Tropical Medicine, Nuffield Department of Medicine, University of Oxford, Oxford, United Kingdom; Universidade Federal de Minas Gerais, Brazil

## Abstract

**Background:**

*Plasmodium vivax* imposes substantial morbidity and mortality burdens in endemic zones. Detailed understanding of the contemporary spatial distribution of this parasite is needed to combat it. We used model based geostatistics (MBG) techniques to generate a contemporary map of risk of *Plasmodium vivax* malaria in Indonesia in 2010.

**Methods:**

*Plasmodium vivax* Annual Parasite Incidence data (2006–2008) and temperature masks were used to map *P. vivax* transmission limits. A total of 4,658 community surveys of *P. vivax* parasite rate (*Pv*PR) were identified (1985–2010) for mapping quantitative estimates of contemporary endemicity within those limits. After error-checking a total of 4,457 points were included into a national database of age-standardized 1–99 year old *Pv*PR data. A Bayesian MBG procedure created a predicted *Pv*PR_1–99_ endemicity surface with uncertainty estimates. Population at risk estimates were derived with reference to a 2010 human population surface.

**Results:**

We estimated 129.6 million people in Indonesia lived at risk of *P. vivax* transmission in 2010. Among these, 79.3% inhabited unstable transmission areas and 20.7% resided in stable transmission areas. In western Indonesia, the predicted *P. vivax* prevalence was uniformly low. Over 70% of the population at risk in this region lived on Java and Bali islands, where little malaria transmission occurs. High predicted prevalence areas were observed in the Lesser Sundas, Maluku and Papua. In general, prediction uncertainty was relatively low in the west and high in the east.

**Conclusion:**

Most Indonesians living with endemic *P. vivax* experience relatively low risk of infection. However, blood surveys for this parasite are likely relatively insensitive and certainly do not detect the dormant liver stage reservoir of infection. The prospects for *P. vivax* elimination would be improved with deeper understanding of glucose-6-phosphate dehydrogenase deficiency (G6PDd) distribution, anti-relapse therapy practices and manageability of *P. vivax* importation risk, especially in Java and Bali.

## Introduction

*Plasmodium vivax* malaria is the most widely distributed species of human malaria, threatening nearly 3 billion people in 95 countries ranging from temperate to tropical in the Americas, Africa, and Asia [1], [2]. Unlike the other common cause of malaria, *Plasmodium falciparum*, dormant liver stages of *P. vivax* cause relapses of acute malaria [3]. Despite the reputation of *P. vivax* as a benign infection with very low risk of death, contemporary studies demonstrate substantial morbidity [4], [5], [6], [7] and mortality [8], [9], [10], [11] burdens in endemic zones.

Drug resistance and neglect of its research in *P. vivax* exacerbates the threat of this infection. The first line therapy against acute attack, chloroquine, has failed in Indonesia [12], [13] and parts of Oceania [14], and resistance now threatens the Mekong region [15], [16], [17], [18] and the Indian sub-continent [19], where >90% of *P.vivax* malaria occurs [20]. Although several artemisinin combination therapies (ACT) have shown good efficacy against acute *P. vivax* [21], only primaquine can eliminate the hypnozoite reservoir of infection [22], [23]. The safety and efficacy of primaquine, especially when used with an ACT, is virtually unknown in 2012 [24]. The distribution of risk of this infection emerges as a vital consideration in developing strategies that may mitigate this potentially serious threat. This may be especially true in places like the vast number of islands scattered within the Indonesian archipelago, and those with very limited resources for dealing with such problems.

Other nations have developed such maps. Brooker *et al.* [25] developed a *P. vivax* map for Afghanistan in 2006 at a spatial resolution of 8×8 km using logistic regression models and malaria surveys from 269 endemic villages. Manh *et al.* [26] derived a *P. vivax* distribution map in Vietnam for 2010 using zero-inflated Poisson regression models in a Bayesian framework from 12 months of *P. vivax* malaria reported cases from 670 districts. Reid *et al.* [27] produced a *P. vivax* prevalence map on Tanna Island, Vanuatu in 2008 at 1×1 km resolution using 220 geo-referenced villages and the Bayesian geostatistical logistic regression model. Dogan *et al.* [28] developed *P. vivax* malaria incidence maps at 0.4×0.4 km resolution in Turkey using malaria data from 81 provinces for over 34 years (1975–2008) using a kriging method.

This report describes the first use of a Bayesian model-based geostatistics (MBG) approach [29] to predict the risk of *P. vivax* malaria in Indonesia in 2010 at a spatial resolution of 1×1 km, using the largest assembled contemporary empirical evidence for any country in Asia. This collaborative effort between the Ministry of Health in the Republic of Indonesia and the Malaria Atlas Project (MAP, http://www.map.ox.ac.uk) aims to equip those responsible for national planning and implementation of malaria control and elimination strategies with an evidence base for the distribution of risk of vivax malaria in Indonesia.

## Methods

### Assembling a national database of *Plasmodium vivax* Annual Parasite Incidence data

The Sub-Directorate of Malaria Control at the Directorate of Vector-borne Diseases, Indonesia Ministry of Health in Jakarta routinely collected *P. vivax* Annual Parasite Incidence (*Pv*API) at the district level between 2006 and 2008. The reported cases of confirmed *P. vivax* malaria per 1,000 people were computed for each year by district and averaged over the number of reporting years. Each *Pv*API summary estimate was mapped by matching it to its corresponding first (provincial) and second level (district) administrative unit in a geographic information system (GIS; ArcView GIS 9.3, ESRI, 2008).

### Assembling a national database of *Plasmodium vivax* malariometric prevalence

The process of assembling community-based survey parasite prevalence data undertaken since 1985 has been described previously [30]. Data searches for *P. vivax* parasite rate (*Pv*PR) aimed to retrieve data from published and unpublished sources. These searches are an on-going activity of the Malaria Atlas Project (MAP, http://www.map.ox.ac.uk) and were completed for the current iteration on 25 November 2011. The completed database was checked *via* various levels of exclusion criteria in order to obtain the final input data set for modelling as follows: removing surveys located only to large (>100 km^2^) and small polygons (>25 km^2^), removing those surveys that could not be precisely geo-positioned and removing those that could not be temporally disaggregated into independent surveys or for which the survey date was unknown. The entire database was then checked to ensure all survey sites were located precisely on grid squares identified as land and within the border of the country. Finally, the database was checked for any spatio-temporal duplicates. The dataset was then stratified into two regions for descriptive purposes, since western and eastern Indonesia are biogeographically distinct regions of the archipelago, typically demarked by the Wallace Line [31].

### Assembling Indonesia human population data

Gridded population counts and population density estimates at 1×1 km spatial resolution for the years 1990, 1995 and 2000, both adjusted and unadjusted to the United Nations' national population estimates were provided by The Global Rural Urban Mapping Project (GRUMP) *beta* version [32], [33]. The adjusted population counts for the year 2000 were projected to 2010 by applying the relevant national urban and rural growth rates by country [34] using methods described previously [35]. The urban growth rates were applied to populations residing within the GRUMP-defined urban extents [33], and the rural rates were applied elsewhere. National 2010 totals were then adjusted to match those estimated by the United Nations [36]. These population counts were then stratified nationally by age group using United Nations-defined [36] population age structures for the year 2010 to obtain population count surfaces for the 0–5 years, 5–14 years and ≥15 years age groups. This population surface was extracted for Indonesia and aligned to all other spatial data grids used in the analysis.

### Defining the limits of *Plasmodium vivax* transmission

Annual Parasite Incidence data at district level in 33 endemic provinces were sourced to define the spatial limits of *P. vivax* transmission in 2010. Following previously defined protocols [1], classification of risk based on *Pv*API data assigned areas at no risk (zero annual incidence over three years), unstable (mean annual incidence less than 0.1 per 1,000 people per annum) or stable risk (mean annual incidence higher than 0.1 per 1,000 people per annum). These polygon-based data were then rasterised to 1×1 km spatial grids. A temperature mask was then applied on *Pv*API data-defined limits of transmission [29]. This biological mask delineated areas where low temperatures were likely to inhibit parasite development in anopheline vectors [37]. We further modified a decision rule for stable transmission. Within stable transmission limits, pixels predicted with high certainty (probability >90%) of being less than 1% *Pv*PR_1–99_ were downgraded from stable to unstable class. This extremely low prediction was caused by a great abundance of survey data reporting zero prevalence in those areas.

### Environmental covariates

A minimal set of covariates were included to inform prediction of the mean function, based on *a priori* expectations of the major environmental factors modulating transmission. These were (i) an indicator variable defining areas as urban or rural based on the GRUMP urban extent product [32], [33]; (ii) a long-term average vegetation index product as an indicator of overall moisture availability for vector oviposition and survival [38], [39]; and (iii) a *P. vivax* specific index of temperature suitability derived from the same model used to delineate suitable areas on the basis of vector survival and sporogony [37].

### Bayesian space-time geostatistical modelling

Bayesian space-time geostatistical modelling for disease prevalence mapping has been fully described [29] and implemented at the national [40] and global scales [29]. The underlying value of *Pv*PR_1–99_ in 2010, 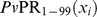, at each location 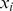 was modelled as a transformation 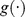 of a spatiotemporally structured field superimposed with unstructured (random) variation 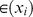. The number of *P. vivax* positive responses 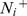 from a total sample of 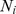 individuals at each survey location was modelled as a conditionally independent binomial variate given the unobserved underlying age-standardized *Pv*PR_1–99_ value [41]. An age-standardisation procedure [42], [43] was implemented to allow surveys conducted in participants of any age range to be converted to the epidemiologically informative 1 to 99 year age range using an algorithm based on catalytic conversion models first adapted for malaria by Pull and Grab [44]. This age-standardisation procedure has been previously adopted for *P. falciparum* [29], [40], but the model form has been reparameterised using assembled age-stratified *Pv*PR surveys. Each survey was referenced temporally using the mid-point (in decimal years) between the recorded start and end months. The spatio-temporal component was represented by a stationary Gaussian process 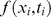 with mean 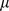 and covariance defined by a spatially anisotropic version of the space-time covariance function proposed by Stein [45]. A modification was made to the Stein covariance function to allow the time-marginal model to include a periodic component of wavelength 12 months, providing the capability to model seasonal effects in the observed temporal covariance structure. These effects arise when studies performed in different years but during similar calendar months have a tendency to be more similar to each other than would be expected in the absence of seasonality. The mean component 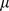 was modelled as a linear function of a vector of the selected suite of environmental covariates, 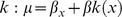. The unstructured component 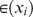 was represented as Gaussian with zero mean and variance 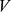. Bayesian inference was implemented using Markov Chain Monte Carlo to generate 100,000 samples from the posterior distribution of: the Gaussian field 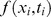 at each data location, the unobserved parameters 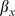, 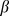, and *V* as stated above and further unobserved parameters defining the structure and anisotropy of the exponential space-time covariance function. Distances between locations were computed in great-circle distance to incorporate the effect of the curvature of the Earth, which becomes important for a nation as large as Indonesia. Samples were generated from the 2010 annual mean of the posterior distribution of 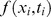 at each prediction location. For each sample of the joint posterior, predictions were made using space-time conditional simulation over the 12 months of 2010 {t = 2010*_Jan_*, …, 2010*_Dec_*}. These predictions were made at points on a regular 1×1 km spatial grid. Model output therefore consisted of samples from the predicted posterior distribution of the 2010 annual mean *Pv*PR_1–99_ at each grid location, which were used to generate point estimates. The uncertainty metric was computed by calculating the ratio of posterior distribution interquartile range to its mean. This standardized metric produced an uncertainty index which less influenced by underlying prevalence levels.

### Evaluating model performance

An empirical model assessment was carried out by first selecting a validation set. Ten percent (n = 445) of the full data points were selected using a spatially de-clustered stratified random sampling algorithm, described previously [29]. Those surveys located outside the stable limits of transmission were excluded from selection. Using the remaining 90% (n = 4,012) of data points the model was then re-run to make predictions at the space-time locations of these held-out data. Model performance was then evaluated using two criteria: the ability of the model to (1) predict point-values of *Pv*PR_1–99_ at validation locations, and (2) to generate credible intervals (CI) that capture appropriately the uncertainty associated with predictions at each location.

The ability of model to predict point-values of *Pv*PR_1–99_ at validation locations was then evaluated by comparing observed values to those predicted (using the posterior mean) by the model at the corresponding locations. Assessment was made using three summary statistics [29], [46] including (1) the mean prediction error, (2) the mean prediction absolute error, and (3) the linear correlation coefficient. The mean prediction error measures the bias of prediction and the mean prediction absolute error measures the accuracy of predictions. The correlation coefficient indicates the linear association between predicted and observed values, which was also visualised using a scatter plot [47].

A sample semi-variogram was calculated from standardised model residuals to assess the presence of residual spatial autocorrelation unexplained by the model output. Standardised Pearson residuals were calculated for each validation location [48], [49]. This sample semi-variograms was compared to a Monte Carlo envelope computed from 99 random permutations of the same residual set [50]. Where the semi-variogram of standardized model Pearson residuals lies entirely within this envelope, it can be considered as the absence of spatial structure.

The ability of the model to generate appropriate credible intervals was tested via a coverage plot. Working through 100 progressively narrower credible intervals, from the 99% CI to the 1% CI, each was tested by computing the actual proportion of held-out prevalence observations that fell within the predicted CI. Plotting these actual proportions against each predicted CI level allows the overall fidelity of the posterior probability distributions predicted at the held-out data locations to be assessed.

### Measuring area and population at risk

The quantification of areas within no risk, unstable and stable category was undertaken by first projecting the predicted class map from geographic to Mollweide equal area projection in ArcGIS 9.3. The areas covered by each category were then calculated in km^2^. To derive population at risk within each zone, this categorical map was overlaid with the GRUMP-*beta* 2010 gridded population surface using an exact bespoke algorithm written in Fortran90, and the total population living in each risk category was calculated. These totals were further disaggregated by province level.

## Results

### The spatial limits of *Plasmodium vivax* transmission

**Figure 1:**
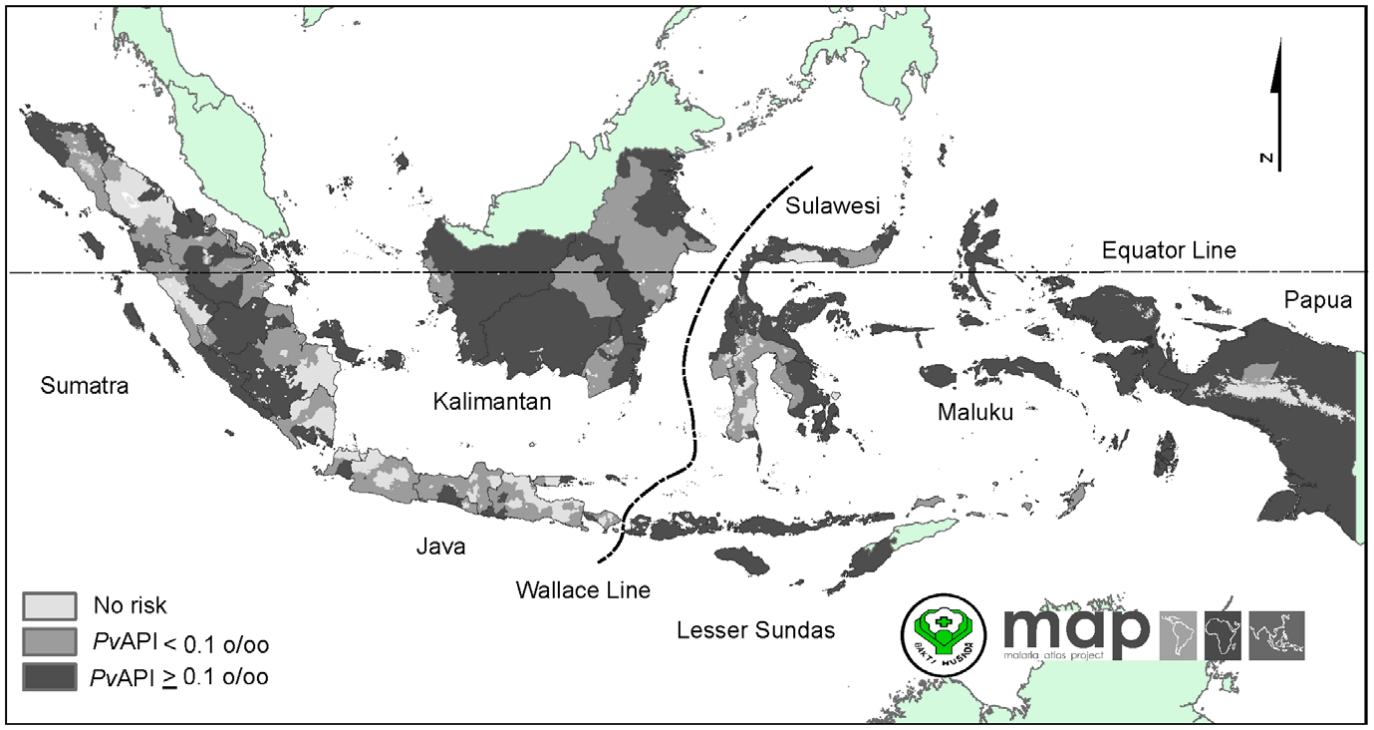
The spatial limits of *Plasmodium vivax* defined by Annual Parasite Incidence and the temperature mask. Areas were defined as stable (dark grey areas, where *Pv*API≥0.1 per 1,000 pa), unstable (medium grey areas, where *Pv*API<0.1 per 1,000 pa), or no risk (light grey, where *Pv*API = 0 per 1,000 pa).

The 2010 *Plasmodium vivax* malaria risk limits in Indonesia are shown in Figure 1. We have estimated that 1.7 million km^2^ (89.8%) of a total land area of 1.9 million km^2^ were endemic for *P. vivax* malaria (Table 1). These endemic areas, a land area of 0.695 million km^2^ (40.7%) were unstable transmission zones and 1.014 million km^2^ (59.3%) were stable transmission zones. Stable vivax transmission zones were more common in eastern than western Indonesia (83.5% vs. 33.7%). Further provincial level estimates of areas at risk are provided in Table S1.

**Table 1:** Area and population at risk of *Plasmodium vivax* malaria in 2010 throughout the Indonesian archipelago.

Area and population at risk	Region				Total	
	Western		Eastern			
	Value	%	Value	%	Value	%
***Area (km^2^)***	***1,153,945***	***100.0***	***748,886***	***100.0***	***1,902,831***	***100.0***
No risk	143,050	12.4	50,261	6.7	193,311	10.2
At risk	1,010,895	87.6	698,625	93.3	1,709,520	89.8
Unstable	922,284	53.9	73,035	9.8	695,319	36.5
Stable	388,611	33.7	625,590	83.5	1,014,201	53.3
***Population***	***204,915,987***	***100.0***	***27,628,308***	***100.0***	***232,544,295***	***100.0***
No risk	96,726,120	47.2	6,157,027	22.3	102,926,147	44.3
At risk	108,146,867	52.8	21,471,281	77.7	129,618,148	55.7
Unstable	96,586,342	47.1	6,176,858	22.4	102,763,200	44.2
Stable	11,560,525	5.7	15,294,423	55.3	26,854,948	11.5

No risk, unstable and stable risk areas correspond to *Pv*API = 0 per 1,000 pa, 0<*Pv*API<0.1 per 1,000 pa and *Pv*API≥0.1 per 1,000 pa.

### Summaries of *P. vivax* malaria prevalence survey data

**Figure 2:**
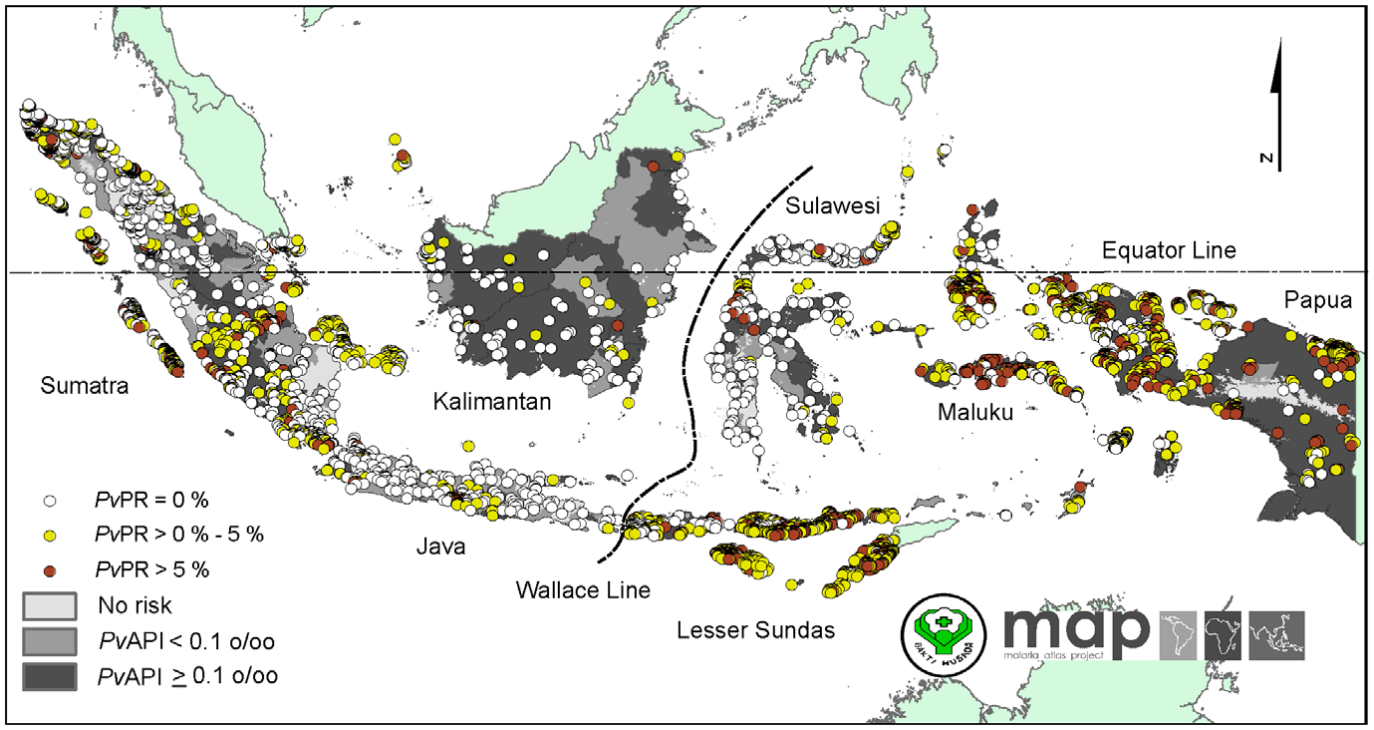
The distribution of *Plasmodium vivax* prevalence surveys in Indonesia between 1985 and 2010. The 4,457 community surveys of *P. vivax* prevalence conducted between 01 January 1985 and 25 November 2011 are plotted. The survey data are shown in white (*Pv*PR = 0%), yellow (*Pv*PR>0%–5%) and red (*Pv*PR>5%). Areas were defined as stable (dark grey areas, where *Pv*API≥0.1 per 1,000 pa), unstable (medium grey areas, where *Pv*API<0.1 per 1,000 pa), or no risk (light grey, where *Pv*API = 0 per 1,000 pa).

A total of 80 different sources from between 1985 and 2010 documented a total of 4,658 independent community surveys of *Pv*PR from 33 *P. vivax* malaria endemic provinces (Figure 2). Provinces of Papua/West Papua (n = 1,021), East Nusa Tenggara (n = 734) and Aceh (n = 434) contributed 47% of total data points. After database fidelity checks, a total of 201 survey locations were excluded from modelling because they were polygon data (n = 6), could not be geo-positioned (n = 87), surveys could not be disaggregated temporally (n = 39), were spatio-temporal duplicates (n = 50) or were missing information on the month of survey (n = 19).

Table 2 summarizes the remaining 4,457 data points *Pv*PR by region. More *Pv*PR surveys were conducted in the eastern region compared to the western region (58% vs. 42%). Over half of the total data points (57.4%) documented the presence of *P. vivax*. In eastern Indonesia, 73.4% of the surveys reported presence records, compared to 35.6% in western region. Mean *Pv*PR was higher in the eastern than the western region (5.6% vs. 1.5%). The great majority of the *Pv*PR data (91.8%) were obtained from unpublished works. Peer-reviewed sources only contributed about 6% of the total data points. A total of 92% of the full number of *Pv*PR records were obtained from direct communication with malaria specialists across Indonesia, the Indonesian National Malaria Control Program and National Health Institute of Research and Development. Most of the data incorporated resulted from *Pv*PR surveys conducted between 2005 and 2010 (88.4%). The great majority of surveys included an upper age >20 years (94.3%). About seven percent of surveys were geo-positioned by Global Positioning Systems (GPS) whilst over 70% of the survey sites were geo-positioned using a combination of paper source, map and geo-referencing techniques. Surveys with small sample sizes (n<50) represented 8.95% of the total data. The median sample size was 136. The most common sample size in western region was 50–100 people (38.8%) whilst in eastern region was 100–500 people (48.9%). The most commonly recorded malaria diagnostic technique in these *Pv*PR surveys was microscopy method (54%).

**Table 2:** Summary of the most important aspects of the *Pv*PR data by main region.

Total records of input data set	Western	Eastern	Total	Percentage
	(n = 1,886)	(n = 2,571)	(n = 4,457)	(100%)
**Number selected for model**				
Population sample size	426,341	955,469	1,381,810	
Number of *Pv*PR>0	672	1,886	2,558	57.39
Mean (standard deviation) *Pv*PR (%)	1.49 (3.97)	5.57 (9.27)	3.84 (7.76)	
Median (range) *Pv*PR (%)	0 (0–45.9)	1.87 (0–86.1)	0.62 (0–86.1)	
**Primary source of ** ***Pv*** **PR data**				
Peer reviewed sources	104	156	260	5.83
Unpublished work	1,688	2,405	4,093	91.83
Reports†	94	10	104	2.34
**Source of spatial coordinates**				
Personal communication	35	39	74	1.66
GPS	129	165	294	6.60
Encarta	235	329	564	12.65
Combination	1,333	1,817	3,150	70.68
Other digital gazettes	112	161	273	6.12
Paper source	4	1	5	0.11
Map	38	59	97	2.18
**Time period**				
1985–1989	99	11	110	2.47
1990–1994	58	60	118	2.65
1995–1999	35	60	95	2.13
2000–2004	81	115	196	4.39
2005–2010	1,613	2,325	3,938	88.36
**Upper age sampled**				
≤10	17	40	57	1.28
>10 and ≤15	70	10	80	1.79
>15 and ≤20	-	117	117	2.63
>20	1,799	2,404	4,203	94.30
**Diagnostic method**				
Microscopy	1,064	1,336	2,400	53.85
RDT	822	1,235	2,057	46.15
**Denominator**				
1–49	272	127	399	8.95
50–100	732	507	1,290	28.94
101–500	572	1,258	1,779	39.91
>500	310	679	989	22.19
Median (Inter Quartile Range; IQR)	95 (65–321)	197 (100–538)	136 (83–450)	

† Ministry of Health reports, theses and other unpublished sources.

The distribution of *P. vivax* malaria surveys was not uniform among the main islands in the archipelago (Figure 2). The islands of Sumatra (western), Papua (eastern) and Lesser Sundas (eastern) were reported as the three richest *Pv*PR data islands with proportions of 32.8%, 22.4% and 19.1%, respectively. Kalimantan was reported as the island with the sparsest *Pv*PR data (3%) followed by Sulawesi (4.6%). In Java, where more districts reported no-risk of vivax malaria, 6.5% of *Pv*PR data were collected between 1985 and 2010.

### The spatial distribution of *Plasmodium vivax* malaria endemicity

**Figure 3:**
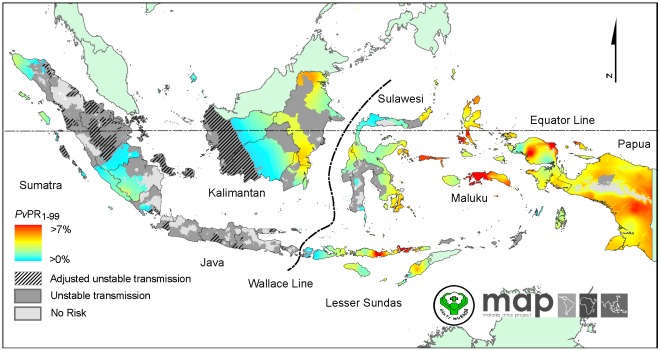
The *Plasmodium vivax* malaria *Pv*PR_1–99_ endemicity map. Model-based geostatistical point estimates of the annual mean *Pv*PR_1–99_ for 2010 within the stable spatial limits of *P. vivax* malaria transmission, displayed as a continuum of light green to red from 0% to 7% (see map legend). Areas within the stable limits in Figure 1 that were predicted with high certainty (>0.9) to have *Pv*PR_1–99_ less than 1% were classified as unstable areas (shaded medium grey areas). The rest of the land area was defined as unstable risk (medium grey areas, where *Pv*API<0.1 per 1,000 pa) or no risk (light grey, where *Pv*API = 0 per 1,000 pa).

**Figure 4:**
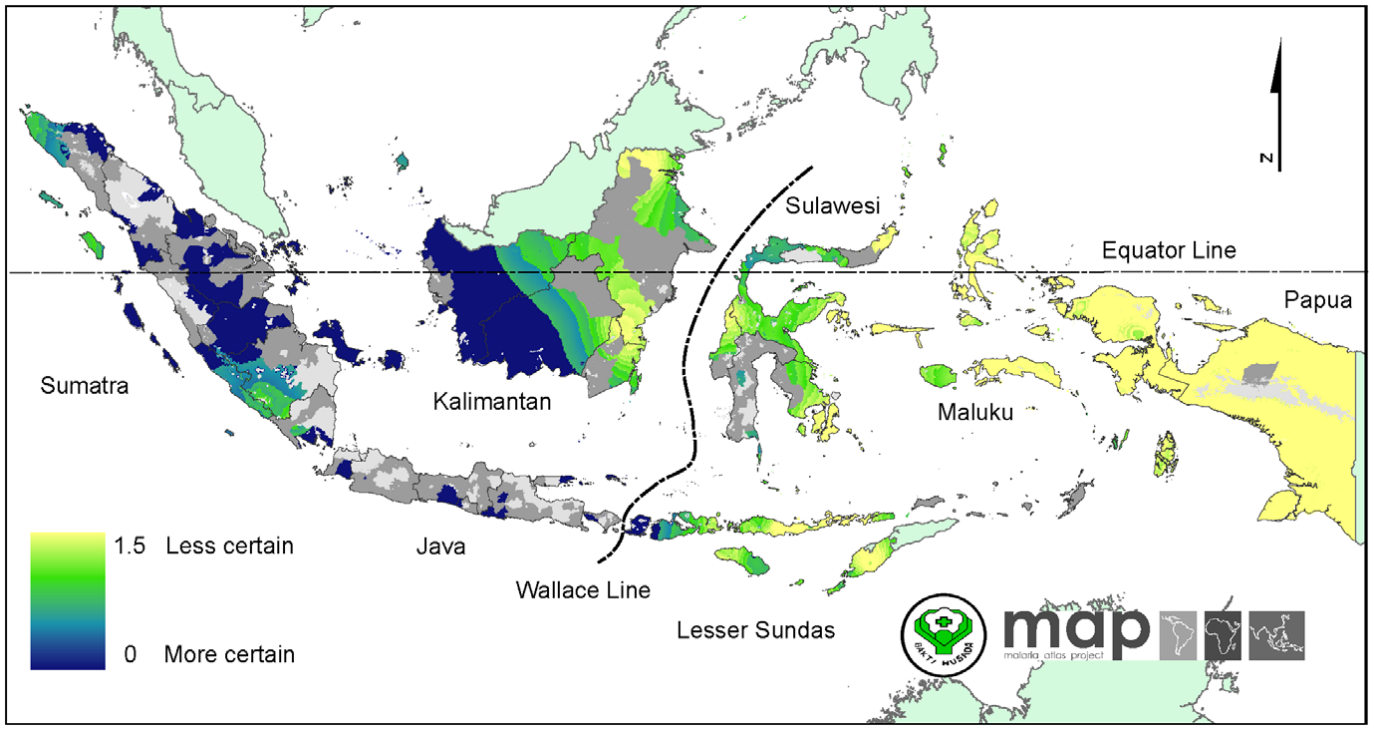
The uncertainty map of predicted *Pv*PR_1–99_ within the stable transmission areas. These values indicate the uncertainty of prediction by using the ratio of posterior inter-quartile range to the posterior mean prediction at each pixel. Large values indicate greater uncertainty. Smaller values indicate higher degree of certainty in the prediction. The rest of the land area was defined as unstable risk (medium grey areas, where *Pv*API<0.1 per 1,000 pa) or no risk (light grey, where *Pv*API = 0 per 1,000 pa).

The continuous predicted surface of *P. vivax* malaria endemicity within the limits of stable transmission is presented in Figure 3. The mean of predicted *Pv*PR_1–99_ was 1.6% with a high degree of heterogeneity ranging from 0.2% to about 11%. In western Indonesia, the predicted *P. vivax* prevalence was uniformly low. Spots of intermediate prevalence *Pv*PR_1–99_ were observed in eastern Kalimantan. High *Pv*PR_1–99_ areas were observed in Lesser Sundas, Maluku and Papua. Uncertainty in predicted *Pv*PR_1–99_ was relatively low in areas with low endemicity and abundance of surveys, such as in parts of Sumatra and Kalimantan (Figure 4). However in areas with high variability of prevalence, such as Papua, certainty of predicted *Pv*PR_1–99_ was relatively lower than other main western islands (Figure 4.).

### The estimation of population at risk of *Plasmodium vivax* malaria

Table 1 shows the estimated population at risk of *P. vivax* malaria in Indonesia in 2010. We have estimated that 129.6 million people (55.7%) lived at risk of *P. vivax* transmission. Of these, 102.8 million (79.3%) and 26.8 million (20.7%) inhabited areas of unstable and stable transmission respectively. Further provincial level estimates of population at risk are provided in Table S1.

In the western region, 108.1 million people (52.8%) live at risk of *P. vivax* transmission.

On Java and Bali islands, (representing 7% of the land area of Indonesia) nearly 77 million people lived in areas of *P. vivax* tranmission, accounting for 71% of all people at risk in western region. More people in western Indonesia lived in unstable transmission than those of stable transmission (89.3% vs.10.7%). The proportion of the population living in unstable versus stable risk was 99% vs. 1% in Java, 63% vs. 37% in Sumatra and 62% vs. 38% in Kalimantan.

In the eastern region, 21.5 million (77.7%) people live at risk of *P. vivax* transmission.

Less people lived in unstable transmission than stable transmission (28.8% vs. 71.2%). All of 10.8 million people lived at risk of *P. vivax* transmission in Sulawesi, followed by 6.7 million in Lesser Sundas, 1.9 million each in both Maluku and Papua. The proportion of the population living in unstable versus stable risk was 49% vs. 51% in Sulawesi, 8% vs. 92% in Maluku, 9% vs. 91% in Lesser Sundas and 3% vs. 97% in Papua.

### Model performance

**Figure 5:**
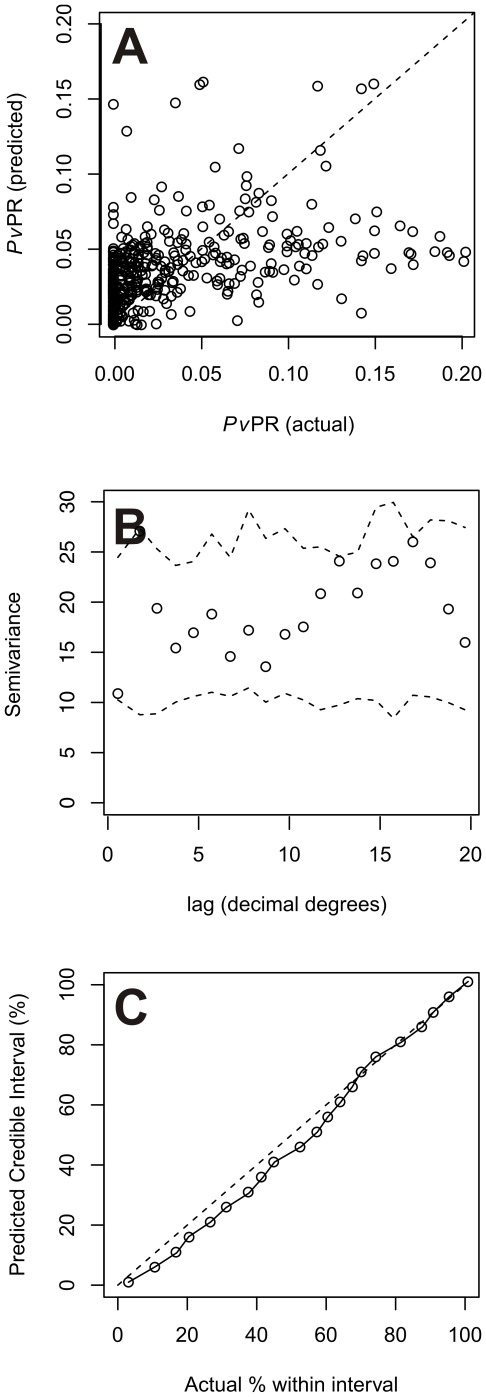
Evaluation of model performance. (A) Scatter plot of actual versus predicted point-values of *Pv*PR_1–99_. (B) Sample semi-variogram of standardized model Pearson residuals estimated at discrete lag and a Monte Carlo envelope (dashed line) representing the range of values expected by chance in the absence of spatial autocorrelation. (C) Probability-probability plot comparing predicted credible intervals with the actual percentage of true values lying inside those intervals. In the top and bottom plots the 1∶1 line is also shown (dashed line) for reference.

In predicting point-values of *Pv*PR_1–99_ at validation locations, the mean prediction error was −0.43% (in units of *Pv*PR_1–99_), indicating low bias in predicted *Pv*PR. This value also represented the tendecy to underestimate *P. vivax* prevalence by just under 0.5%. Mean prediction absolute error, which measured the model precision, was estimated at 3.4% *Pv*PR_1–99_. This value represented the variance between predicted and observed endemicity in each pixel, which is probably due to heterogenity of prevalence in short-range areas or sparsity of data points. The correlation coefficient between predicted and observed values was 0.58, indicating strong linear agreement (see also the corresponding scatter plot, Figure 5A). The semi-variograms of the standardized model Pearson residuals lie entirely within Monte Carlo envelope (Figure 5B) which indicated no significant spatial structure. Figure 5C shows the coverage plot comparing predicted to actual credible intervals. The plotted line is close to the ideal 1∶1 line throughout the range indicating that predicted credible intervals provided an appropriate measure of model uncertainty.

## Discussion

This report describes the spatial limits and level of endemicity of *Plasmodium vivax* in Indonesia. The continous surface *P. vivax* malaria endemicity maps at 1×1 km spatial resolution were generated from an evidence base of nearly 4,500 independent estimates of *P. vivax* malaria prevalence across this archipelago and the use of a Bayesian model-based geostatistical spatial-temporal platform, similar to that applied for *P. falciparum* [29], [40]. These estimates of area and population at risk of *P. vivax* represent improved and updated estimates from those made for 2009 [1]. The detection of *P. vivax* using the Rapid Diagnostic Tests (RDTs) accounted for 46% of assembled malaria prevalence surveys. RDTs are known to be less sensitive than expert microscopy and molecular detection, especially at low parasite densities, which tends to result in higher false-negative rates and, thus, lower observed *Pv*PR [51], [52], [53], [54]. However, precise quantitative adjustments for these factors are not readily available and we have not assessed the impact of this low sensitivity of RDTs on our endemicity estimates.

### Indonesian challenges to control and elimination

Options for malaria preventive measures [55] to reduce the risk of *P. vivax* malaria in Indonesia are limited. No contemporary work has demonstrated the impact of district or nationwide implementation of larvicides, larvivorous fish, or source reduction by environmental management upon malaria transmission. Low coverage rates of insecticide-treated nets (ITN) and their usage, low proportions of houses with screening in endemic zones, variable practices in personal protection represent obstacles to efforts to eliminate malaria transmission in Indonesia. The challenge is further complicated by the unusually diverse mix of 20 *Anopheles* vectors with varying bionomics [56] and interspersed distributions, as recently shown by comprehensive distribution maps of dominant malaria vectors [57]. Another important problem is the availability of reliable diagnostics, which may currently identify fewer than 20% of malaria attacks [12]. A clinical diagnosis in Indonesia prompts therapy with chloroquine or sulfadoxine-pyramethamine, despite widespread resistance to these drugs by both *P. falciparum* and *P. vivax* malaria [12]. Indonesian authorities acknowledge diagnostics as their primary challenge in malaria control.

In the context of controlling or, especially eliminating endemic *P. vivax*, chemotherapeutic attack on the hypnozoite reservoir of infection may be a key consideration. However, the only drug available for this purpose, primaquine, threatens to potentially seriously harm patients with G6PDd [58]. Laboratory screening of those at risk of this harm is not currently practical as part of routine care in Indonesia. The G6PDd prevalences were documented between 1–8% in this archipelago [59], [60], [61], [62], [63]. Although most authoritative agencies recommend a daily dose of primaquine of 0.5 mg/kg for 14 days [64], especially in Southeast Asia [65], [66], this regimen is relatively threatening without G6PDd screening, and the Indonesian authorities thus recommend 0.25 mg/kg for 14 days [67]. Even this lower dose, however, is potentially dangerous and many providers in Indonesia may be reluctant to prescribe it, much less encourage patients to be fully adherent. It may thus be appreciated that G6PDd constitutes a very significant challenge to the Indonesian authorities striving to achieve their declared elimination goals [68].

This risk map of *P. vivax* malaria in Indonesia provides an evidence base which the Indonesian authorities may refer to when developing strategies for the systematic elimination of malaria transmission. The steep challenges imposed by diagnosis, resistance to chloroquine, and the potential harm caused by primaquine may be rationally considered beyond nation-wide solutions. Instead, the resources required to overcome these challenges may be focused upon specific sites where control measures are most needed or where elimination may be realistically within reach. Further, maps of G6PDd prevalence, and some understanding of the distribution of the most vulnerable variants, may also guide balance of risk and benefit with primaquine strategy, policy and practice brought to bear by the authorities [69].

### Further work

The Malaria Atlas Project developed cartographic techniques to estimate clinical burden of *P. falciparum* malaria by using a continuous relationship model between paired *P. falciparum* prevalence and clinical incidence [70], [71]. A non-parametric Bayesian inference was chosen to define this relationship [71]. Space-time joint simulation was then used to measure uncertainty of these clinical burden estimates [70]. In order to achieve similar estimates for *P. vivax*, further work is needed to resolve the association between prevalence of *P. vivax* and clinical incidence. This is especially challenging with the added dimension of relapse and further clinical attacks from a single infectious event. Nonetheless, such estimates constitute vital evidence in rational allocation of limited resources in a nation facing multiple infectious disease threats to the public health.

A glance at the geography of Indonesia reveals yet another challenge faced by the authorities in realizing and maintaining the elimination of malaria from any given island. People from the heavily populated islands of Java and Bali represent a significant proportion of those engaged in the economic development of the many sparsely populated outer islands of the archipelago and it is unknown how many travel back and forth between these islands. These movements incur substantial risk of importing and re-establishing malaria transmission on Java and Bali. MAP and its Indonesian partners will explore techniques to estimate specific patterns and numbers of human movements among the islands in order to identify specific and high priority threats to elimination. The feasibility of such exploration has been facilitated by the advance of geographical information systems, spatial statistics, and anonimized mobile phone records [72], [73], [74] allowing for the tracking of movement of mobile phones among the communications masts that serve them.

### Conclusions

The maps presented in this report constitute part of a suite of GIS tools aimed at providing the authorities in Indonesia responsible for malaria contol with evidence-based means of focusing their resources to where they are most needed and may be most effectively applied. Maps of endemicity of both important species of parasite, coupled with estimates of population at risk and clinical burden, along with the geographic distribution of G6PDd prevalence and patterns of internal migration compose that envisioned suite.

## Supporting Information

Table S1 **Areas and population at risk of** ***Plasmodium vivax*** **malaria in Indonesia by province, main islands and region level in 2010.**
(DOCX)
