# Supplementary material for: Plasmodium vivax Malaria Endemicity in Indonesia in 2010
Source: PLoS One. 2012 May 17;7(5):e37325. doi: 10.1371/journal.pone.0037325 (PMC3355104; doi:10.1371/journal.pone.0037325)
Supplement: Table S1 — Areas and population at risk of Plasmodium vivax malaria in Indonesia by province, main islands and region level in 2010. (DOCX) [file pone.0037325.s001.docx]

Table S1. Area and population at risk of *Plasmodium vivax* malaria in Indonesia by province, main islands and region level in 2010.

| Province | Risk area (km^2^) | | | | | | | | | Total area (km^2^) | | | Population (people) | | | | | | | | | Total population (people) | | |  |  |
| --- | --- | --- | --- | --- | --- | --- | --- | --- | --- | --- | --- | --- | --- | --- | --- | --- | --- | --- | --- | --- | --- | --- | --- | --- | --- | --- |
|  | No risk | | | Unstable | | | Stable | | |  |  |  | No risk | | | Unstable | | | Stable | | |  |  |  |  |  |
|  |  | | |  | | |  | | |  | | |  | | |  | | |  | | |  | | |  |  |
| **Western** | **143,050** | | | **622,284** | | | **388,611** | | | **1,153,945** | | | **96,769,120** | | | **96,586,342** | | | **11,560,525** | | | **204,915,987** | | |  |  |
|  |  | | |  | | |  | | |  | | |  | | |  | | |  | | |  | | |  |  |
| ***Sumatra*** | ***93,689*** | | | ***259,269*** | | | ***123,553*** | | | ***476,511*** | | | ***22,543,764*** | | | ***14,707,432*** | | | ***8,476,821*** | | | ***45,728,017*** | | |  |  |
| Aceh | 4,783 | | | 27,678 | | | 24,607 | | | 57,068 | | | 786,431 | | | 1,224,321 | | | 1,863,916 | | | 3,874,668 | | |  |  |
| Sumatra Utara | 35,594 | | | 31,357 | | | 4,899 | | | 71,850 | | | 9,729,105 | | | 2,690,212 | | | 637,299 | | | 13,056,616 | | |  |  |
| Sumatra Barat | 12,941 | | | 27,729 | | | 1,125 | | | 41,795 | | | 2,833,041 | | | 1,593,423 | | | 13,881 | | | 4,440,345 | | |  |  |
| Riau | 1,618 | | | 88,418 | | | 0 | | | 90,036 | | | 893,711 | | | 3,095,482 | | | 0 | | | 3,989,193 | | |  |  |
| Kepulauan Riau | 0 | | | 6,059 | | | 2,962 | | | 9,021 | | | 0 | | | 1,135,788 | | | 83,402 | | | 1,219,190 | | |  |  |
| Jambi | 1,243 | | | 19,108 | | | 29,078 | | | 49,429 | | | 748,227 | | | 800,072 | | | 975,454 | | | 2,523,753 | | |  |  |
| Bengkulu | 494 | | | 267 | | | 20,363 | | | 21,124 | | | 251,027 | | | 12,786 | | | 1,278,565 | | | 1,542,378 | | |  |  |
| Sumatra Selatan | 23,536 | | | 26,237 | | | 36,396 | | | 86,169 | | | 3,461,870 | | | 1,151,292 | | | 2,712,551 | | | 7,325,713 | | |  |  |
| Bangka Belitung | 357 | | | 15,944 | | | 9 | | | 16,310 | | | 164,738 | | | 739,871 | | | 84 | | | 904,693 | | |  |  |
| Lampung | 13,123 | | | 16,472 | | | 4,114 | | | 33,709 | | | 3,675,614 | | | 2,264,185 | | | 911,669 | | | 6,851,468 | | |  |  |
|  |  | | |  | | |  | | |  | | |  | | |  | | |  | | |  | | |  |  |
| ***Java/Bali*** | ***44,542*** | | | ***93,809*** | | | ***363*** | | | ***138,714*** | | | ***70,511,604*** | | | ***76,865,382*** | | | ***64,770*** | | | ***147,441,756*** | | |  |  |
| Jakarta | 681 | | | 0 | | | 0 | | | 681 | | | 12,523,487 | | | 0 | | | 0 | | | 12,523,487 | | |  |  |
| Banten | 3,334 | | | 6,053 | | | 0 | | | 9,387 | | | 8,100,547 | | | 1,988,513 | | | 0 | | | 10,089,060 | | |  |  |
|  | |  | | | | | | | | |  | | |  | | | | | | | | |  | | |  |
| Province | | Risk area (km^2^) | | | | | | | | | Total area (km^2^) | | | Population (people) | | | | | | | | | Total population (people) | | |  |
|  |  | No risk | | | Unstable | | | Stable | | |  |  |  | No risk | | | Unstable | | | Stable | | |  |  |  |  |
|  | |  | | |  | | |  | | |  | | |  | | |  | | |  | | |  | | |  |
| Jawa Barat | | 13,446 | | | 23,716 | | | 0 | | | 37,162 | | | 24,925,194 | | | 17,131,303 | | | 0 | | | 42,056,497 | | |  |
| Jawa Tengah | | 5,490 | | | 28,861 | | | 0 | | | 34,351 | | | 5,846,771 | | | 29,358,083 | | | 0 | | | 35,204,854 | | |  |
| Yogyakarta | | 526 | | | 2,635 | | | 0 | | | 3,161 | | | 1,576,124 | | | 2,153,578 | | | 0 | | | 3,729,702 | | |  |
| Jawa Timur | | 19,605 | | | 28,340 | | | 363 | | | 48,308 | | | 16,906,146 | | | 23,017,327 | | | 64,770 | | | 39,988,243 | | |  |
| Bali | | 1,460 | | | 4,204 | | | 0 | | | 5,664 | | | 633,335 | | | 3,216,578 | | | 0 | | | 3,849,913 | | |  |
|  | |  | | |  | | |  | | |  | | |  | | |  | | |  | | |  | | |  |
| ***Kalimantan*** | | ***4,819*** | | | ***269,206*** | | | ***264,695*** | | | ***538,720*** | | | ***3,713,752*** | | | ***5,013,528*** | | | ***3,018,934*** | | | ***11,746,214*** | | |  |
| Kalimantan Barat | | 1,534 | | | 86,280 | | | 60,229 | | | 148,043 | | | 927,269 | | | 2,581,801 | | | 592,440 | | | 4,101,510 | | |  |
| Kalimantan Tengah | | 370 | | | 70,994 | | | 83,175 | | | 154,539 | | | 56,944 | | | 532,859 | | | 1,170,522 | | | 1,760,325 | | |  |
| Kalimantan Selatan | | 999 | | | 17,176 | | | 19,408 | | | 37,583 | | | 1,382,456 | | | 1,253,595 | | | 639,413 | | | 3,275,464 | | |  |
| Kalimantan Timur | | 1,916 | | | 94,756 | | | 101,883 | | | 198,555 | | | 1,347,083 | | | 645,273 | | | 616,559 | | | 2,608,915 | | |  |
|  | |  | | |  | | |  | | |  | | |  | | |  | | |  | | |  | | |  |
| **Eastern** | | **50,261** | | | **73,035** | | | **625,590** | | | **748,886** | | | **6,157,027** | | | **6,716,858** | | | **15,294,423** | | | **27,628,308** | | |  |
|  | |  | | |  | | |  | | |  | | |  | | |  | | |  | | |  | | |  |
| ***Sulawesi*** | | ***21,621*** | | | ***54,524*** | | | ***112,085*** | | | ***188,230*** | | | ***4,577,945*** | | | ***5,345,801*** | | | ***5,516,072*** | | | ***15,439,818*** | | |  |
| Sulawesi Utara | | 2 | | | 8,009 | | | 6,772 | | | 14,783 | | | 89 | | | 942,211 | | | 1,203,290 | | | 2,145,590 | | |  |
| Gorontalo | | 6,385 | | | 275 | | | 5,499 | | | 12,159 | | | 173,400 | | | 173,575 | | | 498,270 | | | 845,245 | | |  |
| Sulawesi Tenggara | | 1,031 | | | 9,101 | | | 26,987 | | | 37,119 | | | 304,388 | | | 292,971 | | | 1,205,004 | | | 1,802,363 | | |  |
| Sulawesi Barat | | 1,232 | | | 5,063 | | | 10,066 | | | 16,361 | | | 123,083 | | | 457,237 | | | 283,491 | | | 863,811 | | |  |
|  | | |  | | | | | | | | |  | | |  | | | | | | | | |  | | |
| Region/Province | | | Risk area (km^2^) | | | | | | | | | Total area (km^2^) | | | Population (people) | | | | | | | | | Total population (people) | | |
|  |  |  | No risk | | | Unstable | | | Stable | | |  |  |  | No risk | | | Unstable | | | Stable | | |  |  |  |
|  | | |  | | |  | | |  | | |  | | |  | | |  | | |  | | |  | | |
| Sulawesi Tengah | | | 1,890 | | | 483 | | | 58,860 | | | 61,233 | | | 264,188 | | | 129,147 | | | 1,776,471 | | | 2,169,806 | | |
| Sulawesi Selatan | | | 11,081 | | | 31,593 | | | 3,901 | | | 46,575 | | | 3,712,797 | | | 3,350,660 | | | 549,546 | | | 7,613,003 | | |
|  | | |  | | |  | | |  | | |  | | |  | | |  | | |  | | |  | | |
| ***Maluku*** | | | ***202*** | | | ***9,057*** | | | ***69,754*** | | | ***79,013*** | | | ***1,431*** | | | ***147,241*** | | | ***1,805,662*** | | | ***1,954,333*** | | |
| Maluku | | | 196 | | | 9,057 | | | 37,705 | | | 46,958 | | | 1,347 | | | 147,241 | | | 1,073,103 | | | 1,221,691 | | |
| Maluku Utara | | | 6 | | | 0 | | | 32,049 | | | 32,055 | | | 84 | | | 0 | | | 732,559 | | | 732,643 | | |
|  | | |  | | |  | | |  | | |  | | |  | | |  | | |  | | |  | | |
| ***Lesser Sundas*** | | | ***1,038*** | | | ***1,018*** | | | ***64,377*** | | | ***66,433*** | | | ***1,355,225*** | | | ***634,739*** | | | ***6,063,506*** | | | ***8,053,470*** | | |
| Nusa Tenggara Barat | | | 999 | | | 1,018 | | | 17,867 | | | 19,884 | | | 1,344,784 | | | 634,739 | | | 2,176,468 | | | 4,155,991 | | |
| Nusa Tenggara Timur | | | 39 | | | 0 | | | 46,510 | | | 46,549 | | | 10,441 | | | 0 | | | 3,887,038 | | | 3,897,479 | | |
|  | | |  | | |  | | |  | | |  | | |  | | |  | | |  | | |  | | |
| ***Papua*** | | | ***27,400*** | | | ***8,436*** | | | ***379,374*** | | | ***415,210*** | | | ***222,426*** | | | ***49,077*** | | | ***1,909,183*** | | | ***2,180,685*** | | |
| Papua | | | 26,105 | | | 8,436 | | | 283,322 | | | 317,863 | | | 213,471 | | | 49,077 | | | 1,375,616 | | | 1,638,164 | | |
| Papua Barat | | | 1,295 | | | 0 | | | 96,052 | | | 97,347 | | | 8,955 | | | 0 | | | 533,567 | | | 542,522 | | |
|  | | |  | | |  | | |  | | |  | | |  | | |  | | |  | | |  | | |
| **Indonesia** | | | **193,311** | | | **695,319** | | | **1,014,201** | | | **1,902,831** | | | **102,926,147** | | | **102,763,200** | | | **26,854,948** | | | **232,544,295** | | |
